# 

**Published:** 1896-10

**Authors:** 


*We positively cannot send premium to any subscriber who does not pay a year in advance.*

NOTICE.—Our offer of a thermometer to *all* cash-in-advance subscribers is withdrawn. *New* subscribers, paying full year in advance, may still get the thermometer. Book offer still open to all who pay a year in advance. "Stories of a Country Doctor."

### TRI-STATE MEDICAL SOCIETY OF ALABAMA, GEORGIA, AND TENNESSEE.

The eighth annual meeting of this society to be held in Chattanooga, Tenn., on October 13, 14, and 15, promises to be both interesting and instructive. A number of papers are already on the list, and a large attendance is hoped for. Dr. J. B. Murfree, Murfreesboro, Tenn., is president, and Dr. Frank Trester Smith, Chattanooga, is secretary.

